# Supplementary material for: Endoplasmic reticulum visits highly active spines and prevents runaway potentiation of synapses
Source: Nat Commun. 2020 Oct 8;11:5083. doi: 10.1038/s41467-020-18889-5 (PMC7546627; doi:10.1038/s41467-020-18889-5)
Supplement: Supplementary file 1 — Supplementary Information [file 41467_2020_18889_MOESM1_ESM.pdf]

Supplementary Information

Endoplasmic reticulum visits highly active spines and prevents runaway potentiation of synapses

Perez-Alvarez A. et al.

This pdf file includes:

- Supplementary Figure 1
- Supplementary Figure 2
- Supplementary Figure 3
- Supplementary Figure 4
- Supplementary Figure 5
- Supplementary Figure 6
- Supplementary Figure 7

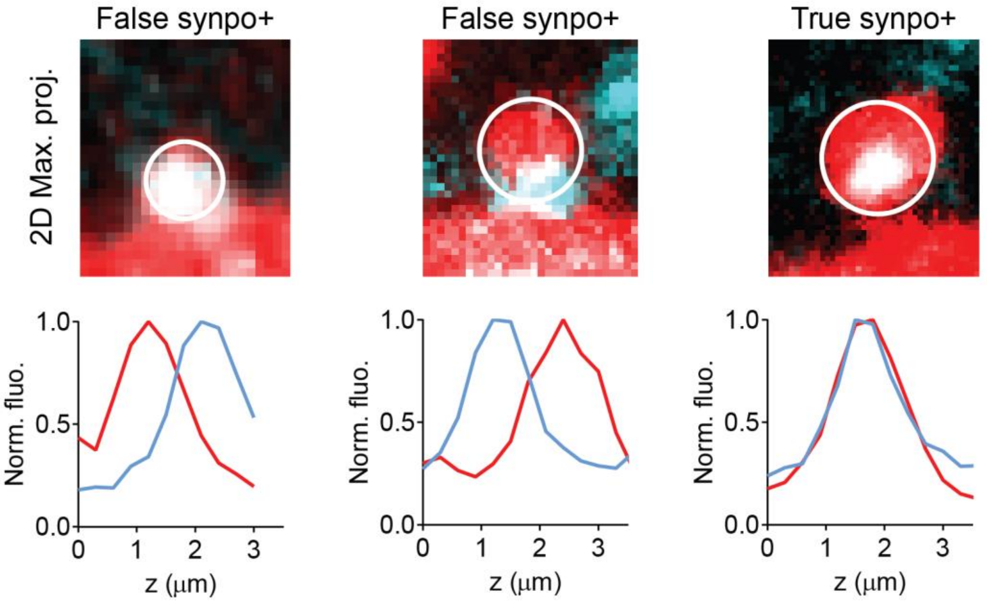

Supplementary Figure 1 | Scoring of the spine apparatus marker synaptopodin in fixed tissue. 2D projections of confocal image z-stacks (upper row) are not sufficient to identify synaptopodin-positive (synpo+) spines. To identify true synpo+ spines, we evaluated the axial intensity profile in confocal image stacks (lower row) in a circular region of interest (white circle). Only spines where an intensity maximum of the anti-synaptopodin signal (cyan) was found in the focal plane of the spine head (red) were scored as synpo+ (right example).

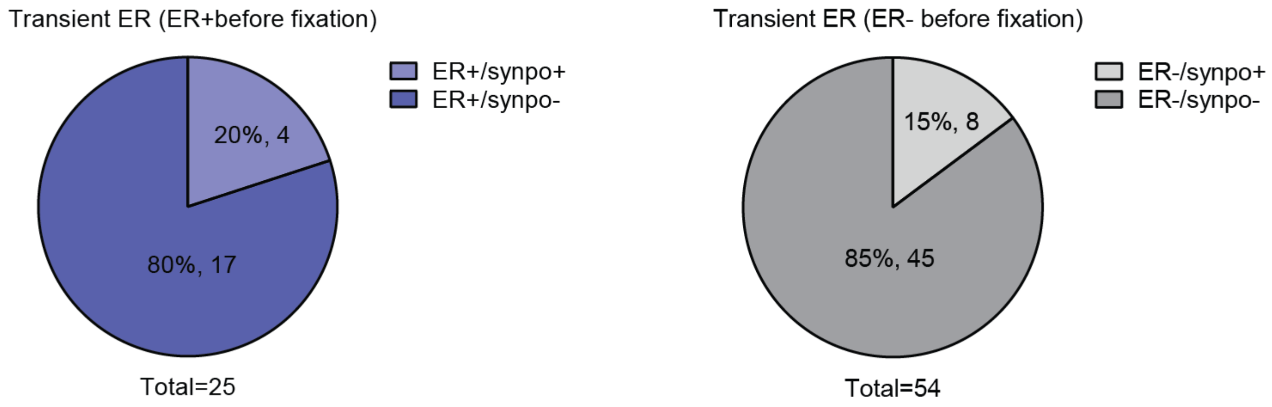

Supplementary Figure 2 | Spines with transient ER are rarely immunoreactive for synaptopodin. To test whether spines that were just invaded by ER were more likely to contain synaptopodin, we split the sample of transient ER spines into the ones that contained ER (ER+, blue) at the last live imaging time point (left diagram) and the ones that did not contain ER (ER-, gray). In both groups, a small percentage was immunoreactive for synaptopodin (20%; 15%).

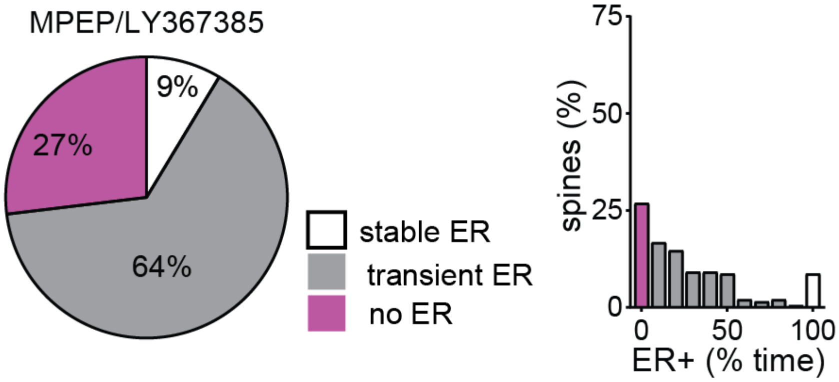

Supplementary Figure 3 | Block of group I mGluRs increases ER motility. Blockade of group I mGluRs with a cocktail of MPEP (50 µM) and LY367385 (100 µM) increased the percentage of spines visited by ER (gray sector) and prolonged visit duration (*n* = 197 spines, 3 cells, 3 slices, $p < 0.0001$, two-sided Mann-Whitney test) compared to controls (see Fig. 1i). Observation time = 100 min.

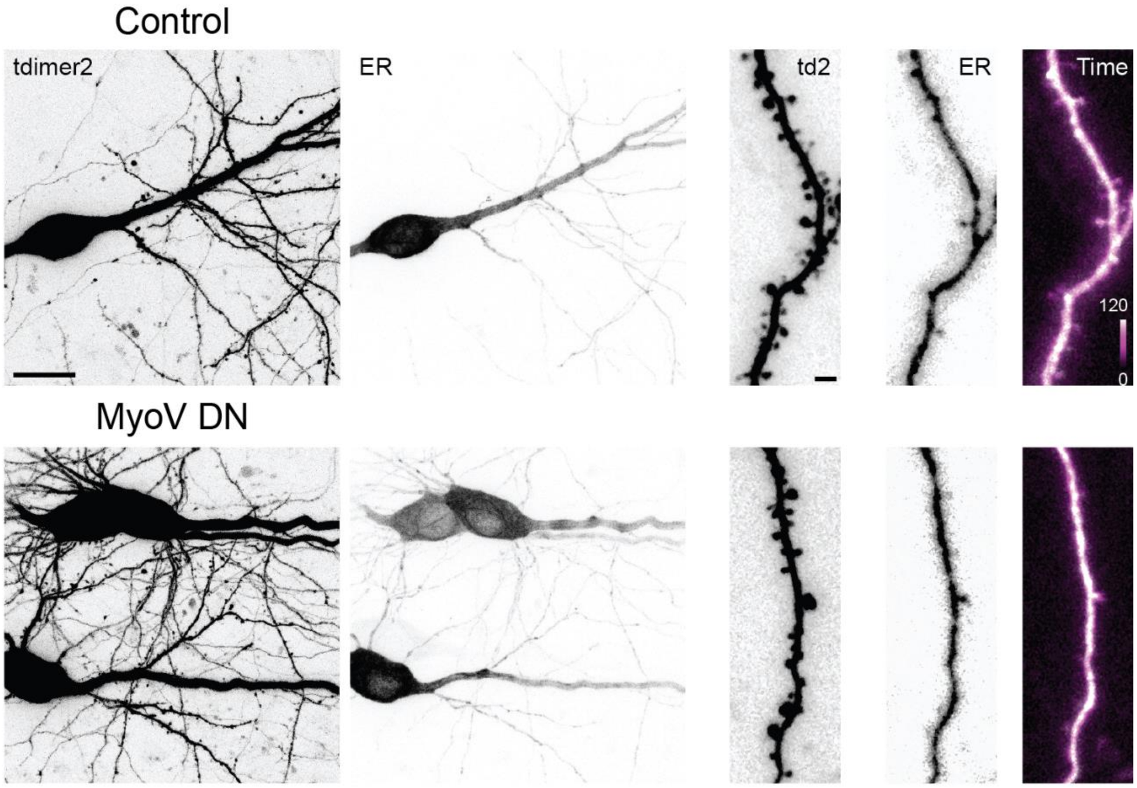

Supplementary Figure 4 | Expression of a MyoVa dominant-negative construct in CA1 neurons. Maximum projections of two-photon z-stacks of tdimer2 (neuron filler) and ER-EGFP from electroporated CA1 neurons expressing fluorescent reporters (above) or also MyoV DN (below). Scale bar: 20 µm. Detail of dendritic segments (right). Scale bar: 2 µm. Collapsed time projection (far right) of a 120 min time lapse, color coded for the duration of ER signal presence. Duration: 120 min. Experiments were replicated in 4 + 4 organotypic cultures (MyoV DN + controls).

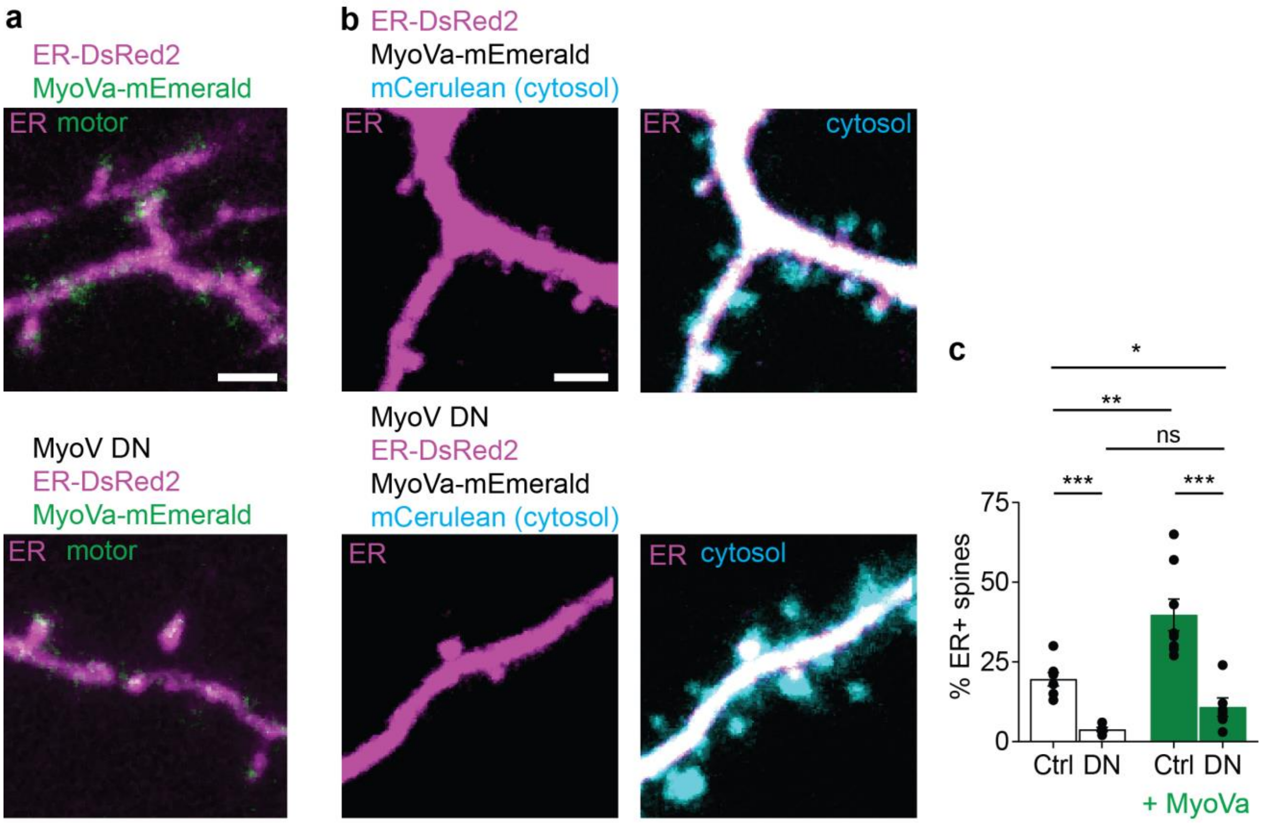

Supplementary Figure 5 | Overexpression of MyoVa shows bidirectional modulation of spine ER.
a, Expression of MyoVa-mEmerald shows the localization of the motor at the tip of ER protrusions in dendrites from control (upper image) and MyoV DN-expressing (lower image) CA1 neurons. For these experiments, we labeled the ER with a red fluorescent protein (ER-DsRed2, displayed as magenta). Scale bar: 2 µm. Experiments were repeated 5 times. **b,** In neurons overexpressing MyoVa-mEmerald, the dominant negative construct MyoV DN (lower images) still had a strong effect on the number of ER+ spines. Cerulean was used as a cytosolic filler to visualize all spines. Scale bar: 2 µm. Experiments were repeated 5 times. **c,** Comparing data (mean ± *SEM*) from Fig. 3c (white bars: control (n = 7 cells, 7 slices) vs MyoV DN (n = 5 cells, 5 slices) , 20 ± 2% vs 4 ± 1% ER+ spines, p = 0.0001, two-sided unpaired t test) and MyoVa-mEmerald overexpression (green bars: control (n = 8 cells, 5 slices) vs MyoV DN (n = 6 cells, 4 slices), 40 ± 5% vs 11 ± 3% ER+ spines, p = 0.0007, two-sided unpaired t test). Overexpression of MyoVa-mEmerald doubled the number of ER+ spines under control conditions (p = 0.004) and tripled the number of ER+ spines in cells expressing the dominant negative construct (p = 0.06). Neurons expressing both MyoVa-mEmerald and the DN construct still have fewer ER+ spines compared to neurons with unperturbed motors (partial rescue, p = 0.03). For statistical analysis, we used two-tailed unpaired t tests.

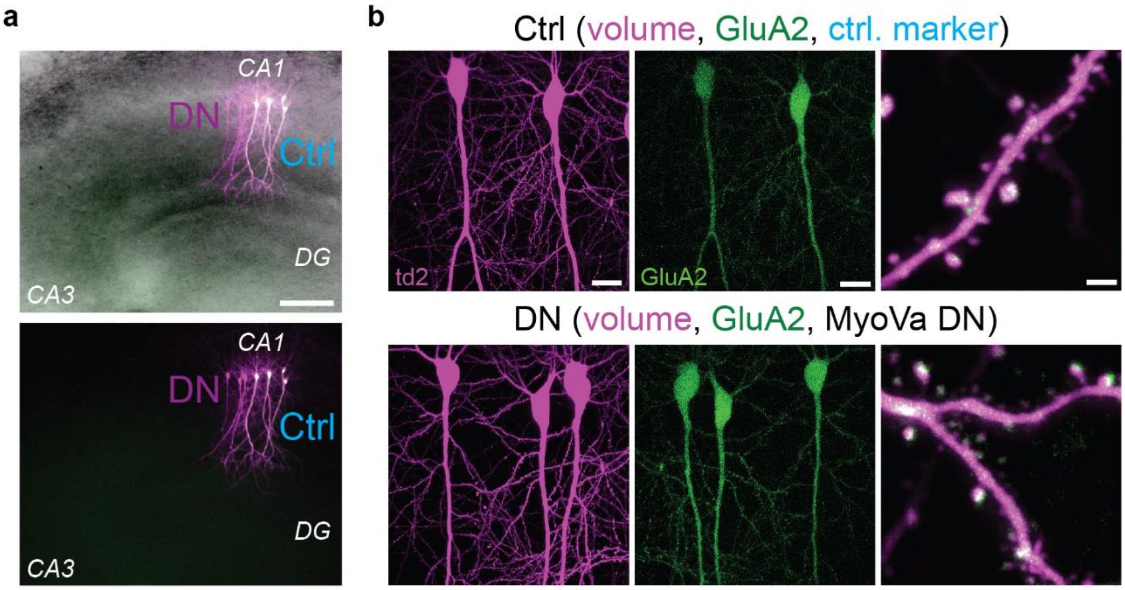

Supplementary Figure 6 | Surface expression of AMPA receptors is enhanced in MyoV DN neurons.
a, Overview of organotypic slice culture, showing control neurons expressing SEP-GluA2, cytosolic tdimer2 and cytosolic mCerulean (white), and DN neurons expressing SEP-GluA2, cytosolic tdimer2 and MyoV DN (magenta). Scale bar: 200 µm. This experiment was repeated on 4 slices. **b**, Volume channel (magenta), SEP-labeled AMPA receptors (green) and high magnification overlay (SEP-GluA2 signal appears white). Upper row: control neurons; lower row: MyoV DN neurons. For quantification, see Fig. 4a, b. Scale bar left and middle panels: 20 µm. Scale bar right panels: 2 µm.

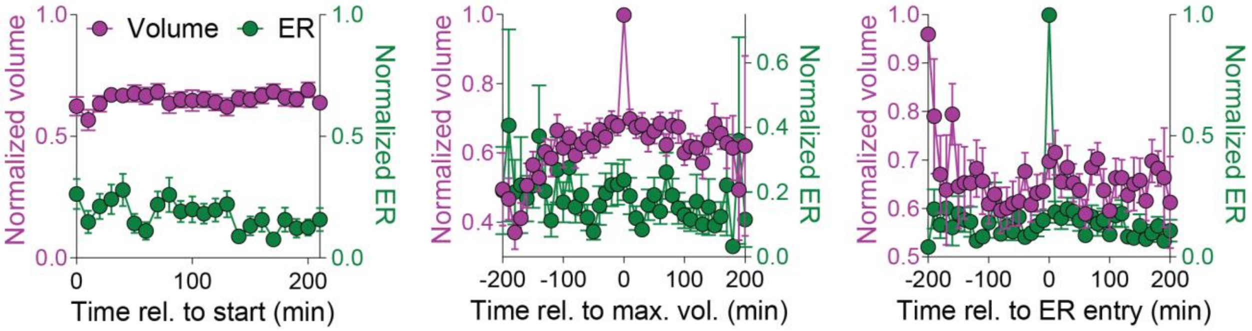

Supplementary Figure 7 | ER calcium depletion abolishes correlation between ER entry and spine volume fluctuations. Thapsigargin (1 µM, 60 min preincubation time), a SERCA pump blocker, preserved ER dynamics and spine volume expansion, but abolished any temporal coupling (*n* = 28 spines from 2 slices). *Left,* average of spine volume and ER fluorescence in spines over time. *Center,* at times of maximum spine volume (t = 0), the probability of spines containing an ER was average. *Right,* At the time of ER visit (t = 0), spine volume was average. These experiments provide evidence that the observed temporal coupling of events in the absence of thapsigargin (Fig. 2e, f) was not an imaging artifact. Data are mean ± *SEM.*
