# Supplementary material for: Endoplasmic reticulum visits highly active spines and prevents runaway potentiation of synapses
Source: Nat Commun. 2020 Oct 8;11:5083. doi: 10.1038/s41467-020-18889-5 (PMC7546627; doi:10.1038/s41467-020-18889-5)
Supplement: Supplementary file 2 — Description of Additional Supplementary Files [file 41467_2020_18889_MOESM2_ESM.pdf]

Description of Additional Supplementary Files

File Name: Supplementary Movie 1

Description: ER dynamics in a dendritic branch of a CA1 pyramidal cell. Maximum intensity projections from two-photon z-stacks (980 nm excitation) taken every 10 min over a period of 5 h. *Upper image*: ER-EGFP signal showing the ER lumen. *Lower image:* ER EGFP signal (green) merged with the cytosolic filler tdimer2 (red). Vertical structure with dim ER signal is an axon.

File Name: Supplementary Movie 2

Description: Uncaging of MNI-glutamate in zero Mg^{2+} induces ER entry into spines. Two-channel time lapse image (*left:* ER-EGFP, green; *right:* cytoplasmic tdimer2, red) of two-photon glutamate uncaging on dendritic spines. Note strong volume increase in stimulated spine and ER entry.

File Name: Supplementary Movie 3

Description: MyoV DN impairs ER entry into spines. Two-photon time lapse showing ER dynamics in a dendritic branch of two CA1 pyramidal cells. *Upper image*: Control neuron, expressing ER-EGFP, tdimer2 and mCerulean. *Lower image*: Neuron with compromised myosin V function (expressing ER-EGFP, tdimer2 and MyoV DN). Note absence of ER visits to spines.
