# Supplementary material for: Endoplasmic reticulum visits highly active spines and prevents runaway potentiation of synapses
Source: Nat Commun. 2020 Oct 8;11:5083. doi: 10.1038/s41467-020-18889-5 (PMC7546627; doi:10.1038/s41467-020-18889-5)
Supplement: Supplementary file 6 — Reporting Summary [file 41467_2020_18889_MOESM6_ESM.pdf]

nature research

Corresponding author(s): *Double-blind peer review submissions: write DBPR and your manuscript number here instead of author names.*

Last updated by author(s): *YYYY-MM-DD*

# Reporting Summary

Nature Research wishes to improve the reproducibility of the work that we publish. This form provides structure for consistency and transparency in reporting. For further information on Nature Research policies, see our Editorial Policies and the Editorial Policy Checklist.

## Statistics

For all statistical analyses, confirm that the following items are present in the figure legend, table legend, main text, or Methods section.

| n/a | Confirmed | |
|---|---|---|
| ☐ | ☒ | The exact sample size ($n$) for each experimental group/condition, given as a discrete number and unit of measurement |
| ☐ | ☒ | A statement on whether measurements were taken from distinct samples or whether the same sample was measured repeatedly |
| ☐ | ☒ | The statistical test(s) used AND whether they are one- or two-sided<br>*Only common tests should be described solely by name; describe more complex techniques in the Methods section.* |
| ☒ | ☐ | A description of all covariates tested |
| ☐ | ☒ | A description of any assumptions or corrections, such as tests of normality and adjustment for multiple comparisons |
| ☐ | ☒ | A full description of the statistical parameters including central tendency (e.g. means) or other basic estimates (e.g. regression coefficient) AND variation (e.g. standard deviation) or associated estimates of uncertainty (e.g. confidence intervals) |
| ☐ | ☒ | For null hypothesis testing, the test statistic (e.g. $F$, $t$, $r$) with confidence intervals, effect sizes, degrees of freedom and $P$ value noted<br>*Give P values as exact values whenever suitable.* |
| ☒ | ☐ | For Bayesian analysis, information on the choice of priors and Markov chain Monte Carlo settings |
| ☒ | ☐ | For hierarchical and complex designs, identification of the appropriate level for tests and full reporting of outcomes |
| ☐ | ☒ | Estimates of effect sizes (e.g. Cohen's $d$, Pearson's $r$), indicating how they were calculated |

*Our web collection on statistics for biologists contains articles on many of the points above.*

## Software and code

Policy information about availability of computer code

| | |
|---|---|
| Data collection | Two-photon imaging and electrophysiology data was acquired using ScanImage (3.8) and Ephus (Matlab-based freeware). |
| Data analysis | For data analysis, we used Fiji, Imaris (Bitplane AG), Matlab (The MathWorks) and Graphpad Prism (v8). Custom Matlab code used for analysis has been deposited at https://gin.g-node.org/Alberto_Perez-Alvarez/Spine_volume_ER_detection. |

For manuscripts utilizing custom algorithms or software that are central to the research but not yet described in published literature, software must be made available to editors and reviewers. We strongly encourage code deposition in a community repository (e.g. GitHub). See the Nature Research guidelines for submitting code & software for further information.

## Data

Policy information about availability of data

All manuscripts must include a data availability statement. This statement should provide the following information, where applicable:

- Accession codes, unique identifiers, or web links for publicly available datasets
- A list of figures that have associated raw data
- A description of any restrictions on data availability

Source data are provided with this paper. Raw data and reagents are available from Thomas G. Oertner (thomas.oertner@zmnh.uni-hamburg.de) on reasonable request.

# Field-specific reporting

Please select the one below that is the best fit for your research. If you are not sure, read the appropriate sections before making your selection.

- [x] Life sciences
- [ ] Behavioural & social sciences
- [ ] Ecological, evolutionary & environmental sciences

For a reference copy of the document with all sections, see nature.com/documents/nr-reporting-summary-flat.pdf

# Life sciences study design

All studies must disclose on these points even when the disclosure is negative.

| | |
|---|---|
| Sample size | Sample size was not predetermined. Regarding ER visits and glutamate uncaging on single spines, we consider each synapse as an independent unit. For evaluation of the effect of MyoV DN expression, we compared two groups of neurons, which represent our 'n' in these experiments. |
| Data exclusions | In electrophysiologcal experiments, recordings in which the series resistance exceeded 20 MOhm were excluded from the analysis. |
| Replication | All data sets were collected over many days, using slice cultures produced from different animals. All experiments were reproducible. For optogenetic plasticity induction, we used two different opsins; results (LTP, LTD) were near identical and data were pooled. |
| Randomization | In all MyoV DN experiments, we compared to control cells expressing fluorescent constructs only. The experimenter was blind to the genoytpe during imaging and analysis. Thus, randomization was achieved by our blinding procedure. |
| Blinding | Blinding was used in this study for comparisons between cells expressing or not expressing MyoV DN (i.e. synaptic plasticity, SEPGluA2 and glutamate uncaging). |

# Reporting for specific materials, systems and methods

We require information from authors about some types of materials, experimental systems and methods used in many studies. Here, indicate whether each material, system or method listed is relevant to your study. If you are not sure if a list item applies to your research, read the appropriate section before selecting a response.

## Materials & experimental systems

| n/a | Involved in the study | |
|---|---|---|
| [ ] | [x] | Antibodies |
| [x] | [ ] | Eukaryotic cell lines |
| [x] | [ ] | Palaeontology and archaeology |
| [ ] | [x] | Animals and other organisms |
| [x] | [ ] | Human research participants |
| [x] | [ ] | Clinical data |
| [x] | [ ] | Dual use research of concern |

## Methods

| n/a | Involved in the study | |
|---|---|---|
| [x] | [ ] | ChIP-seq |
| [x] | [ ] | Flow cytometry |
| [x] | [ ] | MRI-based neuroimaging |

## Antibodies

| | |
|---|---|
| Antibodies used | Anti-Synaptopodin (SE-19) antibody (Sigma S9442).<br>Alexa Fluor 488 goat anti-rat secondary antibody (Life Technologies). |
| Validation | https://www.ncbi.nlm.nih.gov/pmc/articles/PMC2139823/<br>https://www.ncbi.nlm.nih.gov/pmc/articles/PMC6737913/ |

## Animals and other organisms

Policy information about studies involving animals; ARRIVE guidelines recommended for reporting animal research

| | |
|---|---|
| Laboratory animals | Wistar rats, provided by Janvier or Envigo. |
| Wild animals | The study did not involve wild animals. |
| Field-collected samples | The study did not involve samples collected in the field. |
| Ethics oversight | Animal procedures for Wistar rats were approved by the Behörde für Gesundheit und Verbraucherschutz of the City of Hamburg, Hamburg, Germany. |

Note that full information on the approval of the study protocol must also be provided in the manuscript.
